# Differing characteristics of cartilaginous lesions of the larynx

**DOI:** 10.1007/s00405-019-05563-w

**Published:** 2019-07-23

**Authors:** Alfio Ferlito, Kenneth O. Devaney, Antti A. Mäkitie

**Affiliations:** 1 Coordinator of the International Head and Neck Scientific Group, Padua, Italy; 2 Department of Pathology, Allegiance Health, Jackson, MI USA; 3 Department of Otorhinolaryngology - Head and Neck Surgery, University of Helsinki and Helsinki University Hospital, P.O. Box 263, 00029 HUS, Helsinki, Finland; 4 Research Programme in Systems Oncology, Faculty of Medicine, University of Helsinki, Helsinki, Finland; 5 Division of Ear, Nose and Throat Diseases, Department of Clinical Sciences, Intervention and Technology, Karolinska Institutet and Karolinska Hospital, Stockholm, Sweden

**Keywords:** Surgery, Radiotherapy, Metastasis, Pathology, Histology

## Abstract

**Introduction:**

The tissues of the laryngeal region only rarely harbor primary cartilaginous lesions, and squamous cell carcinoma remains the most frequently encountered malignant tumor in this area.

**Materials and Methods:**

We reviewed the salient histological features of cartilaginous laryngeal lesions to provide differential diagnostics and guidelines for distinguishing the benign from the malignant ones.

**Results:**

Cartilaginous neoplasms of the larynx include chondroma and chondrosarcoma. Among the overarching group of all forms of laryngeal sarcoma, chondrosarcoma forms the most common entity in the larynx, followed by rhabdomyosarcoma. Cartilaginous tumors comprise about 0.1%–1% of all laryngeal neoplasms with chondrosarcomas being more frequently encountered than chondromas. Several neoplasms earlier reported as giant-cell tumors of the larynx would most likely, using current terminology, be classified as cases of undifferentiated pleomorphic sarcoma (previously known as malignant fibrous histiocytoma, giant-cell variant) or aneurysmal bone cyst.

**Conclusion:**

When true laryngeal sarcomas do exist, they may prove to be challenging lesions both for the pathologist and the treating clinician. The diagnostic problems are mainly a result of the infrequent exposure of clinicians and diagnosticians to these lesions.

## Chondrometaplasia

### Definition and epidemiology

Among the family of the cartilaginous lesions of the larynx, chondrometaplasia appears to be the most frequently encountered entity. One early autopsy report described an incidence of asymptomatic laryngeal chondrometaplasia somewhere in the range of 1.2–1.7/100,000 people who died of a variety of causes and were subsequently autopsied [1]. This particular human autopsy study was prompted by those authors’ earlier identification of similar chondrometaplastic lesions in both vocal cords of a male orangutan [2]. The incidence of *symptomatic* chondrometaplasia encountered by clinicians may be higher or lower, e.g., a report by Burtner et al. reported that chondrometaplasia was found in 9% of all vocal cord polyps in surgical pathology material reviewed over a 2-year period [2].

### Etiology

The cause of chondrometaplasia is not known with certainty. In one report, almost 80% of symptomatic individuals had previously undergone endotracheal intubation, raising the possibility that this may be a post-traumatic lesion in at least some—perhaps even a majority of—patients [2]. Other patients in this series had been treated with prior received radiotherapy, while yet other patients were smokers. One autopsy study suggested that the histologic features of developing chondrometaplasia proceed through a predictable series of steps. This involves deposition of acid mucopolysaccharides amongst the collagen bundles in the soft tissues of the false vocal cord, followed by a ‘rounding up’ of the appearance of the fibroblast nuclei, then leading to an appearance of myxoid stromal change in the region, and finally culminating in a proliferation of fibroelastic cartilage [1].

### Pathology

The gross specimen of chondrometaplasia resembles the commonly encountered vocal cord polyp, in that it is typically small (less than one centimeter in diameter), round to oval, and covered by mucosa; this mucosa is most often intact, without ulceration. On cut section, a glistening, gray-white nodule is found in the center of the lesion—an appearance which contrasts with that of the soft, red-tan, fibrovascular core which usually forms the center of the more commonly encountered vocal cord polyp.

By light microscopy, chondrometaplastic lesions are immediately recognizable as cartilaginous in nature, as indicated by their arrangement of isolated cells residing within lacunae, which are widely spaced in a basophilic or eosinophilic matrix. The individual nuclei are small, single, homogenously dark, and lack mitotic activity. The key histological feature of chondrometaplasia, however, relates not to the individual cells which form the lesion but rather to the elastic cartilage matrix in which they are embedded [3].

In addition, a gradual blending of the elastic cartilage stroma into the surrounding fibrous stroma is often seen by light microscopy. This finding contrasts with the better defined, sharp distinction between the hyaline cartilage of a true chondroma and its surrounding stroma. This “transition zone” in chondrometaplasia is marked by a contrast between centrally located rounded nuclei situated within lacunae and the progressively flattened cells, more reminiscent of fibroblast nuclei, found at the periphery of the lesion.

Aside from the use of histochemical staining for elastin to highlight the elastic cartilage nature of the matrix, it is unlikely that a surgical pathologist will be forced to rely upon ancillary diagnostic techniques, such as immunohistochemical or ultrastructural studies, to confirm a diagnosis of chondrometaplasia.

### Clinical features

Laryngeal chondrometaplasia appears both in symptomatic patients typically complaining of a change in their phonation, and in asymptomatic patients (in this instance, usually discovered at the time of clinical evaluation of an unrelated problem or at autopsy). Those patients who complained of hoarseness reported having had symptoms for an average of 7 months prior to clinical examination [2]. The incidence of chondrometaplasia seems to be slightly greater in males than in females. Chondrometaplasia is, in general, a lesion of adulthood; however, although it has not been reported in patients during the first decade of life, it has been described in patients ranging in age from the second decade of life to the tenth, with an average age at presentation of around 52 years [2, 4–7]. In the majority of cases, chondrometaplasia arises in the vocal cords and less often in the epiglottic region [3, 7, 8].

The lesions may be solitary or multiple and may affect one or both sides of the larynx. Symptomatic lesions usually occupy the region of the true vocal cords followed in incidence by the epiglottis and the ventricle. By contrast, incidentally discovered asymptomatic lesions of laryngeal chondrometaplasia are usually located in the area of the false vocal cords.

Pathologists are familiar with the finding of metaplastic changes within laryngeal tissues, e.g, they commonly encounter often witness squamous metaplasia in the respiratory epithelium, or osseous metaplasia in cartilage (the latter is often a concomitant of aging and may be seen in the structures composed of hyaline cartilage, including the cricoid cartilage, thyroid cartilage and the body of the arytenoid cartilage).

### Diagnosis and differential diagnosis

Symptomatic laryngeal chondrometaplastic lesions are usually biopsied at the time of direct laryngoscopy and submitted as “vocal cord polyps”. The asymptomatic lesions are more often encountered by the surgical pathologist at the time of dissection of a laryngectomy specimen resected for another process (most often squamous carcinoma), or at autopsy. Whether single or multiple, the nodules of chondrometaplasia rarely exceed 1 cm in greatest dimension.

Recognition of both the small size of the lesions and their elastic stroma will aid in distinction of these lesions from laryngeal chondromas and chondrosarcomas. Care should be taken not to confuse chondrometaplasia with the normal elastic cartilaginous components of the larynx. Of substantial assistance to the surgical pathologist in this regard is the clinical identification by the surgeon of the nodules of chondrometaplasia as an abnormal growth within the laryngeal region. The epiglottis, corniculate cartilage and cuneiform cartilage are composed of elastic cartilage but are unlikely to be confused with nodules of chondrometaplasia. In addition, the vocal process of the arytenoid is also composed of elastic cartilage and its nodular appearance may invite comparison with chondrometaplasia. The arytenoid vocal processes are sharply circumscribed nodules of elastic cartilage and are not marked by the transition zone (from elastic cartilage to adjacent stroma) often found in lesions of chondrometaplasia. Moreover, the normal arytenoid vocal process is unlikely to be submitted by a surgeon as a “polyp” in a patient with hoarseness.

### Treatment and prognosis

Chondrometaplasia is a benign, presumably reactive process. It has neither been shown to have a metastatic potential nor has it been shown to be a direct cause of death in a given patient. Local excision is the treatment of choice. No premalignant potential has been ascribed to this lesion. Occasional recurrences have been reported but re-excision appears to be curative in these instances [7]. It is for this reason—the typical clinical course—that chondrometaplasia should be separated from the neoplastic chondromas and chondrosarcomas, as the latter two lesions are more likely to behave in a more aggressive fashion than chondrometaplasia. Of course, in the view of the proposed etiology of the lesion—that of a reactive process—it is conceivable that, should the inciting cause (such as trauma for example) persist following excision of a nodule of laryngeal chondrometaplasia, recurrent lesions may well continue to develop; in practice however, this does not appear to be a very common occurrence (Table 1).

**Table 1:** Key features of a laryngeal chondrometaplasia

Relatively common (perhaps as many as 9% of symptomatic vocal cord polyps, 1.2–1.7% at autopsy)	
Symptomatic i.e. change in phonation (vocal cords, epiglottis, ventricle), or asymptomatic (false vocal cords)	
Adults (average age 52 years, symptomatic patients)	
Size usually less than 1 cm in diameter	
Elastic cartilage; small homogeneous nuclei, no mitoses	
Differential diagnosis—chondroma	
Transition zone (chondroid center, spindle periphery)	
Recurrences rare following excision, no metastatic potential	

## Chondroma

### Definition and epidemiology

Chondromas are benign mesenchymal tumors—true neoplasms, and not reactive processes. Earlier reports of laryngeal chondromas may have included some lesions better classified as laryngeal chondrometaplasia and others which probably represented low-grade chondrosarcomas. Nevertheless, benign proliferations of hyaline cartilage do occur in the larynx and so, a true chondroma may enter the surgical pathologist’s differential diagnosis of both chondrometaplasia and low-grade chondrosarcoma.

A critical review of chondroma cases is virtually impossible, because most reports lack pathological documentation and follow-up data. Chondroma of the larynx is considered uncommon and occurs most frequently in males, usually in middle age. Traves is credited with the first description of this lesion in the larynx in 1816 [9]. Accounts of cases in which a diagnosis of primary chondroma was followed by the diagnosis of chondrosarcoma for the recurrent tumor raise the very real possibility that (supposing some degree of confusion between benign chondromas and low-grade chondrosarcomas might have existed) the incidence of chondroma may well have been been overestimated, making true laryngeal chondromas extremely rare [10]. Only two cases of laryngeal chondroma were seen at the Mayo Clinic between 1910 and 1979 among a total of 33 cartilaginous tumors of the larynx [11]. Of the 323 cases of benign neoplasms of the larynx reviewed from the pathology files of the Eye and Ear Hospital and Presbyterian University Hospital of Pittsburgh, dating from 1955 to 1984, only two cases were observed [12]. Several cases reported as chondromas have been subsequently described as chondrosarcomas. This, in turn, suggests that chondroma and chondrosarcoma either are capable of appearing synchronously in the same tumor or metachronously through a malignant transformation from chondroma to chondrosarcoma; as is often the case in other low-grade mesenchymal tumors—atypical lipomatous tumor (low-grade liposarcoma), e.g., it is likely that a single tumor may show both areas recognizable as low-grade chondrosarcoma as well as areas not recognizable in isolation as malignant, which speaks loudly to the need for thorough sampling of cartilaginous tumors of the larynx.

### Etiology

The specific cause of these tumors is not known. Neither trauma (as in some instances of chondrometaplasia) nor therapeutic irradiation (as in some chondrosarcomas) have been implicated in the formation of laryngeal chondromas.

### Pathology

Both biopsy and definitive excision specimens share a common gross appearance, namely, that of proliferation of nodules of homogenous gray-white translucent tissue with a rubbery, slippery texture. Foci of hemorrhage or necrosis should not be found on gross examination.

On low-power light microscopic examination, chondromas usually have a multinodular architecture. The individual tumor cell nuclei show a distinct degree of uniformity, one to another—they are mostly single nuclei with homogenous dark chromatin set in lacunae; cytological nuclear details are largely obscured. It must be conceded that there is a degree of subjectivity in this requirement; it has been found for example, that a minimal degree of cytological atypia in the extraosseous lesions of synovial chondromatosis is not in itself incompatible with a benign diagnosis. However, when dealing with laryngeal lesions the pathologist is best advised to adhere to the rather stricter criteria employed in the recognition of intraosseous chondrosarcomas (i.e., a lower threshold for recognizing atypia as significant), instead of the somewhat more liberal standards sometimes applied to soft tissue lesions such as synovial chondromatosis.

The cellularity of chondroma is lower than that of at least some areas of low-grade chondrosarcomas—that is to say, if chondrosarcomas include some 30 or 40 cells per high-power field in the most cellular areas, then the cellularity of chondromas should fall short of that. It has to be repeated, however, that cellularity and atypia may vary from area to area within a chondrosarcoma, and so the pathologist should not be lulled into a false sense of security by a cursory inspection of a few areas in a cartilaginous lesion. Mitotic figures are not a feature of the typical chondroma which means that finding mitotic figures in a presumed chondroma specimen should prompt reconsideration of the possibility that the lesion might in fact be a chondrosarcoma.

Ideally, a large and intact specimen of chondroma will show relatively broad interfaces of chondroid tumor with the adjacent tissues, and an absence of an infiltrative pattern (the latter is more commonly a feature of more aggressive lesions such as a chondrosarcoma). However, this may be difficult to appreciate in specimens consisting of a multitude of small disparate tumor fragments.

Islands of chondroid tumor may be partially surrounded by enveloping bands of thin bony trabeculae; the cells forming these osseous trabeculae are cytologically benign, and so confusion with osteosarcoma should not arise. Arising as, presumably, a degenerative phenomenon, islands of chondromatous tissue may undergo central calcification or ossification. These processes are not accompanied by cytologically malignant cells and should thus not invite confusion with sarcomatous degeneration in an otherwise benign chondroma.

As the cartilaginous nature of this lesion is readily recognized with routinely stained histological preparations, it is unlikely that the surgical pathologist will rely on ancillary diagnostic techniques (such as ultrastructural studies) to confirm a diagnosis of chondroma. It remains to be established whether other methods or markers associated with proliferative activity (such flow cytometry, or immunohistochemical staining for an antigen such as proliferating cell nuclear antigen) will aid in making the distinction of chondroma from low-grade chondrosarcoma of the larynx.

### Clinical features

The cricoid cartilage is most often the site of origin of laryngeal chondroma, followed in turn by thyroid cartilage, the epiglottis, and the body of the arytenoid [13]. In the case of a particularly large chondroma, it may be difficult to determine the tumor’s site of origin. Laryngeal chondromas are typically symptomatic lesions, presenting with airway obstruction and manifesting as dyspnea or as a mass in the neck region.

### Diagnosis and differential diagnosis

These tumors may be solitary or multiple and can arise from any hyaline cartilage of the laryngeal skeleton. The neoplasm resembles the histology of normal cartilage, but modestly increased cellularity may be seen. In contrast to lesions of chondrometaplasia (whose small size usually precludes their detection via imaging studies), chondromas may be studied radiographically to gauge their size and extent prior to surgical exploration. Plain films often disclose the presence of a homogeneous soft tissue density, which may show areas of punctate or ring-like calcifications. These calcifications may be highlighted on CT scans and MR scans, studies which may aid in precisely delineating the extent of the tumor.

Biopsy may be complicated by the rubbery-hard consistency of the chondroid lesion, which may resist gentle attempts at harvesting tissue for diagnosis. The following two key differential diagnostic possibilities are deserving of consideration whenever a pathologist is considering the diagnosis of laryngeal chondroma—one benign, the other malignant. Chondrometaplasia has been confused in the past with true chondromas of the larynx. However, when symptomatic, chondrometaplasia is usually localized to the vocal cords (in contrast to the chondromas, which most often appear in the vicinity of the cricoid cartilage), and it consists of an elastic cartilaginous matrix (as opposed to the hyaline cartilage of a true chondroma). The other differential diagnostic consideration—from the other end of the biological spectrum—is that of laryngeal chondrosarcoma. Some chondrosarcomas are high-grade lesions and easily recognized as malignant and thus, do not pose a major diagnostic problem. The low-grade chondrosarcomas, however, are notorious—both within the bone as well as in an extraosseous site such as the larynx—for the frequency with which they are misdiagnosed by the unwary as benign proliferations. Of some aid in addressing this problem in intraosseous sites is the radiographic appearance of the lesion, but such radiographic clues are not always as helpful in a laryngeal lesion. This will place an even greater burden on the surgical pathologist to interpret the tissue fragments correctly.

For the sake of completeness, there is one final differential diagnostic consideration, albeit a singularly rare one. Hamartomas—seen most often in pediatric patients, less often in adults—may be encountered in the larynx [14–17]. In its broadest sense, the term hamartoma refers to a space occupying lesion composed of normal mature cells/tissues arranges in an abnormal architecture. Not true neoplasms, hamartomas are regarded as developmental abnormalities. As a matter of light microscopy, an (exceedingly rare) laryngeal hamartoma with cartilaginous differentiation might be expected to incorporate mature cartilage, respiratory epithelium, muscular tissue, or other tissues—albeit in an unusual architecture, the chief tip-off to a diagnosis of hamartoma.

In summary, key features of the histology of low-grade chondrosarcoma should be excluded before settling on a diagnosis of chondroma. Specifically, a chondroma should not show evidence of nuclear enlargement, atypia, or mitotic activity. The (subjective) finding of increased cellularity should also prompt consideration of the possibility of a low-grade chondrosarcoma, and so force the pathologist to search for other histological clues (such as cytology and mitotic activity) on which to base a final diagnosis. While some have attached some ominous significance to a finding of binucleate cells in a chondroid lesion, this change alone may be encountered in benign lesions and should not be relied on a marker for malignancy. Foci of myxoid change in the cartilaginous stroma, while not themselves diagnostic of malignancy, are certainly more commonly seen in malignant than in benign lesions. As a result, their identification in what otherwise is apparently a chondroma should prompt the pathologist to investigate the biopsy specimen more closely for cytological features that may indicate that the lesion has potential for more aggressive behavior.

As a general principle, the pathologist should be wary of making a diagnosis of chondroma in any incompletely sampled lesion of the larynx. Should a biopsy yield only tissue fragments of a cartilaginous lesion that lacks the histological features of malignancy, it would be wise to ensure that the specimen in question is not simply an incisional biopsy from a much larger lesion. In such a setting—a limited sampling of a larger cartilaginous lesion in which malignant features are not found—one diagnosis, which may be entertained is that of a “cartilaginous lesion of the larynx, not further subclassified”. This might be accompanied with a comment stating that, while malignant features cannot be found in the present lesion, sampling of the entire lesion (if excision is technically possible) is recommended for precise classification.

The aforementioned considerations will no doubt invite some criticism from the attending surgeon, who would desire a firm diagnosis in order to plan surgical therapy. However, the pathologist can only remind the clinicians that examining one small snapshot from of a complex, heterogeneous lesion will not always predict the appearance of other (yet unsampled) regions, which means that one may be dealing with an occult low-grade malignancy which has not yet been adequately sampled. Variation in the histological pattern within the same lesion—from areas diagnostic of chondroma to areas diagnostic of chondrosarcoma—is a recognized phenomenon, which should enhance the pathologist’s natural suspicion regarding the appearance of any portion of a tumor, which has not yet been exhaustively sampled. As a general rule, the larger the lesion the more likely it is to be malignant.

### Treatment and prognosis

Chondromas are more amenable to surgical resection than are the low-grade chondrosarcomas arising in the same region. One potential complication of surgical therapy is postoperative laryngeal stenosis; stenosis usually follows a more extensive resections, particularly of cricoid lesions. It is in view of this potential complication of extensive therapy (as well as the potential for compromising the anatomic integrity of the larynx, thus forcing the surgeon to perform a total laryngectomy) that the surgeon will understandably be reluctant to perform more extensive surgery than is absolutely necessary. This will, predictably, bring additional pressure to bear on the pathologist to make a binding distinction of benign from malignant at the time of biopsy sampling of a larger lesion. True laryngeal chondromas may attain sizes of a few centimeters in diameter, but do not metastasize (Table 2).

**Table 2:** Key features of a laryngeal chondroma

Rare lesions. May be less common than laryngeal chondrosarcoma, but the true incidence is difficult to judge from older reports in which the distinction from chondrometaplasia and low-grade chondrosarcoma may sometimes have been imperfect	
Symptomatic lesions cause airway obstruction or a palpable mass in the neck	
Usually found in the vicinity of the cricoid cartilage, thyroid cartilage, epiglottis, and arytenoids	
May be a few cm in diameter and usually smaller than a laryngeal chondrosarcoma	
Hyaline cartilage, small, monomorphous nuclei without significant nuclear details, no mitoses	
No transition zone (in contrast to chondrometaplasia)	
Differential diagnosis: chondrometaplasia, low-grade chondrosarcoma (and, most rarely, laryngeal hamartomas)	
May recur following excision, but this is uncommon, no metastatic potential	

## Chondrosarcoma

### Definition and epidemiology

Chondrosarcomas are malignant mesenchymal tumors typified by the elaboration of a chondroid matrix by the malignant tumor cells. Chondrosarcomas are most often thought of as skeletal tumors—they comprise some 20% of skeletal malignancies. By contrast, chondrosarcomas are rarely encountered in the head and neck; when they do arise in the head and neck region, the larynx is one of the most frequently affected sites. In the past, chondroma and chondrosarcoma have sometimes been grouped under the unifying category of “cartilaginous tumors”; in modern practice, however, it is critically important to distinguish the two neoplasms as well as the morphological variants of chondrosarcoma because their biological behaviors are actually quite divergent. In the older literature laryngeal chondroma was more commonly encountered than chondrosarcoma, whereas the application of current diagnostic criteria makes the existence of convincing reports of true laryngeal chondromas quite a rarity.

New was the first to use the term 'chondrosarcoma of the larynx' in 1935 [18]. A host of terms have since been applied for this neoplastic lesion, such as non-calcifying chondrosarcoma, malignant chondroma, dedifferentiated chondrosarcoma, chondrosarcoma with malignant mesenchymal component, extraskeletal chondrosarcoma, and extra-osseous chondrosarcoma (Table 3).

**Table 3:** The differential diagnosis of chondrometaplasia and chondroma of the larynx

	Chondrometaplasia	Chondroma
Symptoms	Hoarseness or asymptomaticr	Obstruction of the airway or swelling of the neck
Gross presentation	Small nodule < 1 cm in diameter	Multilobular tumour
Gross presentation	Ventricular fold and vocal cord	Cricoid and thyroid cartilages
Type of cartilage	Elastic	Hyaline (usually)

Arriving at an accurate estimation of the incidence of laryngeal chondrosarcoma is seriously hampered by the fact that in the early reports describing cartilaginous tumors in this region, low-grade chondrosarcomas were almost certainly mistaken for benign chondromas in some instances. Still, chondrosarcoma (and not fibrosarcoma, as was thought to be the most common laryngeal sarcoma by some observers in the past) appears to be the most frequent sarcoma of the larynx. According to the review by Rinaldo et al. [19] approximately 600 cases were reported in the literature up till 2000. The review by Chin et al. tabulated 592 published cases [20]. The North-American SEER data report by Dubal et al. include 143 cases between 1973 and 2010 [21]. Thompson and Gannon reported 111 cases diagnosed between 1970 and 1997 registered in the Otorhinolaryngology—Head & Neck Tumor Registry of the Armed Forces Institute of Pathology [22]. The largest series belongs to the Armed Forces Institute of Pathology, Washington DC with 240 cases registered from 1929 to 1999 [23]. Table 4 summarizes the largest series (series with more than five cases) of laryngeal chondrosarcoma reported in the world.

**Table 4:** The largest ( > 5 cases presented) published series of laryngeal chondrosarcoma

Series	No. of cases	Period	Authors	Year	Remarks
University Hospital Heidelberg, Germany	7	2013–2018	Akbaba et al. [44]	2018	These cases were treated upfront with raster-scanned carbon ion Radiotherapy
Johns Hopkins, School of Medicine, Baltimore, USA	6	2004–2013	Karatayli-Ozgursoy et al. [45]	2016	All were of cricoid origin, three out of six had a recurrence
Guy’s and St Thomas’ NHS Foundation Trust, London, UK	5	1996–2012	Stavrakas et al. [46]	2016	
Dept. of ORL, Turin, Rome, Belluno, Italy	6	2006–2013	Damiani et al. [47]	2014	All had conservative surgical approach
Centro Hospitalar e Universitário de Coimbra, Portugal	6	2002–2012	Oliveira et al. [48]	2014	One had high grade, five underwent total laryngectomy
Montpellier University Hospital, France	7	2001–2008	Pelliccia et al. [49]	2014	All had low-grade cricoid chondrosarcoma
University of Brescia, Italy	16	2001–2013	Piazza et al. [41]	2014	Eleven were of low grade and five of intermediate grade
Moffitt Cancer Institute USA	5	2004–2011	Jackson et al. [50]	2013	Four were of low grade and one of intermediate grade
Tel Aviv University, Israel	6	1959–2010	Buda et al. [51]	2012	Recurrence developed in two patients 2 and 8 years after primary treatment
Harvard Medical School USA	11	2002–2011	Friedman et al. [52]	2012	^b^
Harvard Medical School, USA	10	1995–2010	Zeitels et al. [53]	2011	Eight underwent conservation function preservation surgery
Croix-Rousse Hospital, France	7	1996–2006	Merrot et al. [54]	2009	All were of low grade
Institut Gustave-Roussy France, and M. D. Anderson Cancer Center, USA	15	1978–1997	Casiraghi et al. [25]	2004	One was of high grade
Wake Forest University, USA	9	1991–2002	Koufman et al. [55]	2004	All were cricoid and of low grade
Armed Forces, Institute of Pathology, USA	111	1970–1997	Thompson and Gannon [22]	2002	Only six were of high grade
Clermont-Ferrand University Medical Center, France	5	1981–1990	Saleh et al. [56]	2002	One was of high grade One was myxoid type and presented with neck metastases
The Royal National Throat, Nose and Ear Hospital, UK	12	1976–1999	Rinaldo et al. [19]	2000	All were of low grade No metastases
Armed Forces Institute of Pathology, USA	240	1929–1999	Dennis K. Heffner personal communication [23]	1999	This series includes the cases of Thompson and Gannon 2002^a^
University Hospital VU, Amsterdam	5	1980–1998	Tiwari et al. [57]	1999	One of the authors previously reported four cases of chondrosarcoma of the cricoid treated from 1985 to 1995 at the same institution. No lymph node or distant metastases
University of Kiel, Germany	5	1975–1995	Lippert et al. [58]	1997	One patient died of brain metastases 3 months after surgery
Mayo Clinic, Minnesota, USA	44	1910–1995	Lewis et al. [59]	1997	All were of low grade. No lymph node or distant metastases^c^
University of Buenos Aires, Medical Center, Argentina	6	1973–1990	Sztern et al. [60]	1993	No lymph node or distant metastases
Mallinckrodt Institute of Radiology, Washington University Medical Center, USA	10	–^d^	Wippold et al. [61]	1993	One was of thyroid origin, all were visible in CT scan
Mount Sinai School of Medicine, New York City, USA	11	1973–1990	Brandwein et al. [26]	1992	Two cases were dedifferentiated chondrosarcoma and one presented with cervical lymph node metastases
Depts. of ORL-HNS, Universities of Brescia (1) Padua (2) and New Haven (3)	8	1983–1989 (1) 1966–1989 (2) 1962–1989 (3)	Nicolai et al. [10]	1990	One case was dedifferentiated chondrosarcoma and the patient presented with lung metastases
Swedish Cancer Registry	6	1958–1972	Östberg et al. [62]	1979	No lymph node or distant metastases
Chevalier Jackson Clinic, USA	10	1935–1970	Al-Saleem et al. [63]	1970	One patient presented with cervical lymph node metastasis
Massachusetts Eye and Ear Infirmary, USA	8	1940–1970	Huizenga and Balogh [64]	1970	One patient presented with lung, kidney, and neck metastases

^a^Thompson and Gannon [22]

^b^This series includes the 10 cases reported by Zeitels et al. [53] for the period 1995–2010

^c^This series includes previous published series (Goethals and Dahlin [65]; Gorenstein et al. [66]; Neel and Unni [11]; Kozelsky et al. [67])

^d^ Six cases were from the series registered during the years 1970–1991 at the Armed Forces Institute of Pathology (AFIP) and therefore, these have been partially reported by Thompson and Gannon [22] and Heffner (personal communication 1999) [23]. Four additional cases with CT examinations were obtained from the teaching archives of the Mallinckrodt Institute of Radiology

### Pathology

#### Gross pathology

The surgical specimen from a laryngeal chondrosarcoma resection specimen may have a highly variable gross appearance, largely as a function of the tumor’s histological grade. The tumor presents as a lobulated submucosal, rounded or oval mass covered by laryngeal mucosa. The neoplasm may be uniformly hard or may have soft and even cystic areas of degeneration. Even in the more advanced stages, the tumor tends to develop within the laryngeal hyaline cartilage and only rarely extends beyond the external perichondrium to invade the surrounding tissues (surrounding tissue invasion is more often found in recurrent lesions). The maximum dimension of the tumor usually ranges 1–6 cm, although massive lesions which the larynx are sometimes observed.

#### Histopathology

Chondrosarcomas can be classified as low-grade (grade I), intermediate-grade (grade II) or high-grade (grade III) chondrosarcomas. Most laryngeal chondrosarcomas are low- or intermediate-grade tumors. The histological pattern of low-grade chondrosarcoma is marked by some degree of overlap with the histologic pattern of a benign chondroma—a distinct pitfall for the unwary pathologist. The neoplasm is composed of a relatively homogeneous stroma marked by basophilic (or, less often, metachromatic) staining with conventional hematoxylin and eosin preparations. In turn, the individual tumor cells—the malignant chondrocytes—are embedded in that chondroid matrix in individual holes in that matrix ("lacunae"). Binucleated and occasionally even multinucleated cells may be found. In at least some areas of the tumor, features including irregular nuclear membranes and prominent nucleoli may be apparent, which serve to buttress a diagnosis of a malignant cartilaginous tumor. Mitoses are usually difficult to find in low-grade chondrosarcomas. Calcification and bone formation are frequent. As noted previously, most laryngeal chondrosarcomas are low-grade (well differentiated) tumors.

Medium-grade chondrosarcomas show increased cellularity, particularly at the periphery of the lobules. The nuclear-cytoplasmic ratio of the chondrocytes is, as a general rule, higher than that seen in benign chondromas and low-grade chondrosarcomas (i.e., cytoplasm is relatively diminished in respect to nucleus size). Plump binucleated cells are also seen. Mitoses, if present, are uncommon.

High-grade chondrosarcomas are rare [24]. This tumor type shows striking cellularity and cytologic atypia, and plump binucleated and multinucleated cells are often seen. The chondrocytes have large nuclei with nucleoli and delicate chromatin, and the nuclear-cytoplasmic ratio is relatively high. Mitoses are frequently seen.

One variant of chondrosarcoma, called dedifferentiated chondrosarcoma, has also been reported in the larynx [25–27] (described by some authors as chondrosarcomas with additional malignant mesenchymal component (CAMMC)) [22]. This term describes a neoplasm in which a high-grade sarcomatous component is associated with a lower-grade cartilaginous component. This is a pattern which echoes the development of other soft tissue tumors, such as dedifferentiated liposarcoma. Two histological components comprise a dedifferentiated chondrosarcoma: islands of low-grade (grade 1 of 3, with foci of grade 2 tumor in some areas) chondrosarcoma, and a second (high grade) component. The high-grade sarcomatous component of a dedifferentiated chondrosarcoma may take the form of an undifferentiated pleomorphic sarcoma (formerly, malignant fibrous histiocytoma), fibrosarcoma, rhabdomyosarcoma, osteosarcoma, or sarcoma (not further classified). The lower grade islands of recognizable cartilaginous tumor are embedded in the high-grade component, with a sharp border separating the two components (rather than a gradual zone of transition). While a minority of the dedifferentiated chondrosarcomas have manifested initially as an apparently low-grade cartilaginous tumor, only showing evidence of a high-grade component at the time of a subsequent recurrence, most of the laryngeal dedifferentiated chondrosarcomas reported to date all appear to have been recognizable as such at the time of initial presentation [26].

Another morphological variant of chondrosarcoma occasionally found in the larynx is chondrosarcoma with extensive myxoid change (characterized by some authors as myxoid chondrosarcoma) [22, 28]. The reason for describing these lesions as chondrosarcomas with extensive myxoid change, and not as myxoid chondrosarcomas, is related to the existence of a well-recognized soft tissue tumor—the extraskeletal myxoid chondrosarcoma. Extraskeletal myxoid chondrosarcomas are characterized by a balanced translocation, t(9;22)(q22;q12); in the absence of a demonstration of such a translocation in a particular laryngeal lesion with the appearances described below, it appears a prudent course to characterize such lesions as "chondrosarcomas with extensive myxoid change".

Chondrosarcomas with extensive myxoid change are characterized by nodular aggregations of relatively small, relatively monomorphous hyperchromatic tumor cells arranged in cords, trabeculae and small nests and set in an abundant basophilic homogenous myxoid material; tumor cell density is often greater at the peripheries of the nodules than in their centers. The tumor cells are usually polygonal with prominent nucleoli, but some spindling may occur as well. Mitotic figures are difficult to find in this tumor. Some minor degree of intracytoplasmic vacuolar change may be apparent, but the multiple large intracellular vacuoles typical of the “physaliferous cells” of a chordoma are not characteristic of a chondrosarcoma with extensive myxoid change. A well-developed plexiform vascular pattern is absent. Recognizable cartilaginous areas may be scanty, and so close attention to the size, shape and architectural arrangement of the tumor cells is essential for the recognition of this tumor.

Clear-cell chondrosarcoma is another rare variant of chondrosarcoma, which may arise in the larynx. Laryngeal clear cell chondrosarcoma is characterized by the presence of large rounded cells with an abundance of clear cytoplasm and prominent nucleoli; often, interspersed multinucleated giant cells and bone trabeculae are present as well. Only a few cases of laryngeal chondrosarcoma have been described in the literature. It is a slow-growing tumor—usually a low-grade tumor—and this necessitates a long-time follow-up of patients to monitor for recurrent disease. Due to the extreme rarity of this tumor in the head and neck region, a diagnosis of clear cell chondrosarcoma in this area should be confirmed by histochemical and immunohistochemical studies [29–35]. To date a laryngeal mesenchymal chondrosarcoma does not appear to have been reported. The histological features of the laryngeal chondrosarcomas are summarized in Table 5.

**Table 5:** Histologic subtypes of laryngeal chondrosarcomas

Low-grade chondrosarcoma (grade 1 of 3) (slight increase in cellularity, nuclear size, and nuclear detail over chondroma; no or rare mitoses)	
Intermediate-grade chondosarcoma (grade 2 of 3) (distinct increase in cellularity, nuclear size, nuclear detail, and cytological atypia over chondroma, mitoses still difficult to find)	
High grade chondrosarcoma (grade 3 of 3) (high cellularity , easily recognized cytological atypia; mitotic figures usually readily identified; areas may be difficult to recognize as cartilaginous, owing to exceedingly high cellularity and anaplasia of tumor cells)	
Dedifferentiated chondrosarcoma (chondrosarcoma with additional malignant mesenchymal component) (CAMMC) (low-grade chondrosarcomatous areas juxtaposed with high-grade sarcomatous area—undifferentiated pleomorphic sarcoma (malignant fibrous histiocytoma), fibrosarcoma, rhabdomyosarcoma, osteosarcoma, or sarcoma not further subclassified)	
Chondrosarcoma with extensive myxoid change (nodules of monomorphous round to spindle cells arranged in cords, trabeculae and small clusters and set in a myxoid matrix; mitotic figures not readily identified) (note: tumors with this appearance that manifest a recipirocal t(9;22) translocation may be related to a recognized soft tissue tumor, the extraskeletal myoxid chondrosarcoma)	
Clear cell chondrosarcoma (the tumor contains a component of balloon-like rounded cells with a predominantly clear cytoplasm and prominent nucleoli, in addition to multinucleated giant cells and bone trabeculae; the balloon-like clear cells are glycogen positive)	

#### Immunohistochemistry

Chondrosarcomas show positive staining for S-100 protein and vimentin. The dedifferentiated and myxoid chondrosarcomas are also positive for S-100 protein.

#### Electronmicroscopy

Malignant chondrocytes will show slightly rounded appearance with short processes and scalloping of the membrane. Binucleated cells are typical with nuclei expressing dispersed chromatin, and occasionally with large nucleoli. Abundant cytoplasm typically contains numerous branching segments of rough endoplasmic reticulum and a prominent Golgi apparatus [36, 37].

### Clinical features

Laryngeal chondrosarcomas are most frequently encountered in patients between the age of 50 and 70, but they may also occur in the young. Chin et al. [20] reported an average age of 62.5 years for laryngeal chondrosarcomas at diagnosis (range 15–93); in a similar vein, Thompson and Gannon [22] found an average age at diagnosis of 64.4 years (range 25–91). The reported male-to-female ratio is 3:1 to 4:1 [20, 22, 38]. The most common site of origin is the cricoid cartilage (75%)—particularly, its posterior lamina. Less frequently, there are reports of laryngeal chondrosarcomas arising in the thyroid cartilage, arytenoid cartilages, epiglottis and accessory cartilages. Rare reports of some chondrosarcomas that appear to have originated in the epiglottis exist, an observation that—if proven to be true—may cast some doubt on the commonly held notion that this tumor arises only in hyaline and not in elastic cartilage. Chondrosarcoma may also involve the hyoid bone, but this is not anatomically a part of the larynx.

Symptoms of a laryngeal chondrosarcoma include progressive hoarseness, dyspnea and dysphagia, varying according to tumor location. Dyspnea will predominate if the neoplasm extends anteriorly into the lumen of the airway. If it develops posteriorly into the pharynx, dysphagia will result. As the neoplasm is usually slow-growing, the patient may over time adapt to progressive narrowing of the airway, until an episode of acute inspiratory dyspnea leads to emergency tracheotomy [39]. When the neoplasm originates from the thyroid cartilage, the major complaint may be the presence of a hard lump in the neck produced by the tumor mass. As described in some cases reported in the literature, the first clinical sign may be vocal cord paralysis, apparently unassociated with other laryngeal or non-laryngeal lesions and thus (initially) subject to misidentification as idiopathic in nature. In these cases, vocal cord paralysis is almost exclusively an early sign of cricoid chondrosarcoma and may be related to involvement of the recurrent nerve or fixation of the cricoarytenoid joint.

On routine images of the neck, the neoplasm usually appears as a multilobulated mass marked by varying degrees of calcification. Computed tomography with contrast medium is useful both for demonstrating the extent of the lesion and for raising the possibility of a cartilaginous tumor in the first place. A lesion with calcified areas, extensively involving one or more conventional cartilage bearing regions that is marked by moderate enhancement on CT post contrast, is typical of a laryngeal cartilaginous tumor. However, there are no consistent, reproducible radiographic criteria to permit differentiation between chondrosarcoma and chondroma.

Magnetic resonance imaging can also demonstrate the lesion within the larynx and has the additional advantage of superior contrast resolution of the neoplasm and paralaryngeal tissues.

Chondrosarcoma of the larynx most often extends to local tissues although lymphogenous and haematogenous metastases have been observed in 10% of the cases reported in the literature [22]. When distant spread does occur, lungs and lymph nodes are most frequently involved. Other metastases have occurred in kidney, spleen, cervical spine [37] and occasionally in the humerus [40].

### Diagnosis and differential diagnosis

Both the submucosal location and the rubbery consistency of laryngeal cartilaginous lesions combine to frustrate attempts at securing an adequate biopsy sampling of cartilaginous lesions. It is advisable to obtain the biopsy sample under general anaesthesia and it may be necessary to perform a preliminary tracheotomy to obtain easier access to the lesion. Laryngeal chondrosarcomas often contain areas with different degrees of differentiation, so that only a generous biopsy sample will provide a reliable picture of the tumor's diagnostic macroscopic/microscopic features. Rare reports of diagnosis of laryngeal chondrosarcoma by way of needle biopsy under CT scan guidance have been published.

The distinction between benign and malignant varieties of cartilaginous neoplasms of the larynx is challenging. Cases reported in the literature as benign chondromas, which were actually laryngeal chondrosarcomas, are not uncommon and a correct diagnosis could not be established until a recurrence or metastases developed. In addition, there is no consensus on the subject of the transformation of chondroma to chondrosarcoma remains questionable, whether a tumor with areas reminiscent of chondroma intermixed with areas recognizable as chondrosarcoma represents a chondrosarcoma arising in a pre-existing chondroma, or whether it a single lesion with varying differentiation? The authors favor the latter interpretation but acknowledge that this dilemma remains open for further research.

To reliably establish a distinction between benign and malignant cartilaginous lesions, attention should be concentrated more on the component of existing cells than on the intercellular matrix. In better-differentiated examples, a malignancy should be suspected in the presence of many cells with plump nuclei, more than just occasional cells with two such nuclei, giant cartilage cells with large, single or multiple nuclei or with irregular clumps of chromatin. Nuclear irregularity and the prominence of nucleoli are additional features suggestive of a malignancy. Mitoses are usually scanty. Lobularity may represent a microscopic feature of malignancy, with the periphery of lobules often marked by high cellularity and cytological irregularities. Proliferative activity of cells (as might be highlighted, for example, by immunohistochemistry with antibody to Ki-67) may provide an aid in the distinguishing of chondroma and highly-differentiated chondrosarcoma. The differential diagnostic possibility of a chondroblastic osteosarcoma warrants consideration; areas of calcification and non-neoplastic bone may be present in chondrosarcoma but, unlike in osteosarcoma, bone formation by the cytologically malignant tumor cells is not seen in chondrosarcoma.

Depending on the light-microscopic appearances of the case, differential diagnoses might also include, fibrosarcoma, myxoid liposarcoma, embryonal rhabdomyosarcoma, spindle cell squamous carcinoma with cartilaginous metaplasia, and chondrometaplasia [6].

### Treatment

Surgical resection is regarded as the treatment of choice for these tumors. According to the current literature close to one-third of the patients undergo total laryngectomy [41]. Radiotherapy does not seem to play an important role, though it has been used in the past for recurrent tumors unsuitable for surgical treatment. A conservative surgical approach (open or endoscopic) is often selected in the case of low-grade chondrosarcoma with the objective of organ preservation [42], but the tumor must be radically removed, with a wide margin of normal tissue, and the adjacent external perichondrium resected [10]. The majority of recurrences observed in the past were probably related to piecemeal excisions sparing the external perichondrium. Supraglottic laryngectomy is indicated only when the lesion is restricted to the supraglottic region [37].

The problem becomes more complex when dealing with cricoid lesions, because this cartilage is considered the keystone in maintaining an adequate laryngeal airway. When the lesion is limited in size and involves less than half of the cricoid, conservative removal through a laryngofissure approach can be employed. Different techniques have been described to reconstruct the laryngeal lumen and to prevent stenosis.

In light of the fact that cervical lymph node metastases are rare, neck dissection should be reserved for cases in which the clinical picture or imaging techniques suggest lymph node involvement. Chemotherapy has not proved to be effective for chondrosarcoma of the larynx.

### Prognosis

Chondrosarcomas carry a wide range of clinical behavior, ranging from locally aggressive non-metastasizing neoplasms occasionally invading the thyroid gland, to high-grade malignancies metastasizing via the blood stream to the lungs. Generally speaking, the clinical course of this neoplasm depends on the tumor's degree of differentiation, extension of growth, as well as on the adequacy of primary treatment. Grade I chondrosarcomas usually show a locally aggressive behavior with a limited propensity for metastasis. Metastases are more typical for grade II and even more so for grade III lesions.

The current WHO Classification [43] states that the 1-year, 5-year, and 10-year disease-specific survival rates for chondrosarcoma are 96.5%, 88,6%, and 84,8%, respectively. Also, Chin et al. [20] report disease-specific survival rates for 1, 5, 10, and 20 years to be 97.7%, 91.4%, 81.8%, and 68.0%, respectively, with no differences when correlating 5-year survival rates to location, grade, and therapy.

Table 6 contains the salient features of laryngeal chondrosarcoma, and Table 7 compares clinical and histopathological features of chondrometaplasia, chondroma and chondrosarcoma.

**Table 6:** Key features of a laryngeal chondrosarcoma

Rare lesions: true incidence difficult to judge, as distinction from chondroma has not always been based on modern criteria	
Usually arise in the vicinity of the cricoid cartilage (less often the thyroid cartilage, epiglottis, and body of the arytenoid)	
Typically large lesions: 1–6 cm in diameter (usually larger than laryngeal chondromas)	
Hyaline cartilage	
Cytological features vary from barely perceptible nuclear atypia to readily apparent anaplasia (as a function of the histological type and grade of the tumor)	
Differential-chondroma	
Recurrence may occur following excision, may metastasize (frequency related to histological type and grade

**Table 7:** Comparison of cartilaginous lesions of the larynx

	Chondrometaplasia	Chondroma	Chondrosarcoma
Presentation	Asymptomatic or hoarseness	Dyspnea, hoarseness, mass	Dyspnea, hoarseness, mass, vocal cord paralysis, dysphagia
Common locations	Vocal cords, epiglottis	Cricoid, thyroid cartilage, epiglottis body of the arytenoid	Cricoid, thyroid cartilage, epiglottis, body of arytenoid
Size	Less than 1 cm	Usually 1–3 cm	Usually over 3 cm
Fibrocartilage	Present	Absent	Absent
Hyaline cartilage	Absent	Present	Present
Stromal myxoid change	Absent	Absent	May be present
Cellularity	Low	Low	Slight increase to tremendous increase
Cytological atypia	Absent	Absent	Minimal to pronounced
Mitotic figures	Absent	Absent	Rare to numerous
Invasive border	Absent	Absent	Present

## Conclusion

Chondrometaplasia, chondroma and chondrosarcoma form the rare entity of laryngeal cartilaginous neoplastic lesions. For differential diagnostic purposes both the clinical information as well as an expert opinion of a head and neck pathologist on an adequate biopsy sample will be needed. Management of these neoplasms should be centralized to experienced centres.
